# Literary Notes

**Published:** 1909-04-03

**Authors:** 


### Literary Notes.

Messrs. Bale, Sons, and Danielsson, Ltd., announce the immediate issue of a History of the Reading Pathological Society, written by the President, Dr. Jamieson B. Hurry. This Society is one of the oldest medical societies in the country, and has accomplished a large amount of work. Numerous original communications have been published during the past seventy years. The volume will be illustrated by a series of portraits, and contain descriptions of the Medical Library and Pathological Museum. This work should prove of interest to the Secretaries of the various local medical societies throughout the United Kingdom, as with one exception—that of the Royal Medical and Chirurgical Society—such a record is believed to be unique.

### MEDICATED SOAPS.

(Messrs. D. and W. Gibbs, Limited, City Soap Works, London, E.)

We have received from this firm samples of fourteen of their medicated soaps. These preparations include antiseptic soaps, which are impregnated with carbolic acid, iodoform, boracic acid, and so on, as well as the more strictly medicated soaps for use in various forms of skin disease. The different drugs used are presented in an excellent state of purity, and have been combined with the soapy ingredients in a thoroughly satisfactory way. They are pleasant to use, and efficient both as cleansers and as a means of obtaining the local action of the particular drug used upon the skin. The different medicated soaps contain respectively: Sulphur, 10 per cent.; white birch tar; ichthyol, 5 per cent., and tar; eucalyptol, 5 per cent.; sulphur, camphor, and balsam of Peru; corrosive sublimate, ½ per cent.; borax, naphthol, and sulphur; boracic acid, 5 per cent.; salicylic acid and sulphur; carbolic acid, 20 per cent.; resorcin, 2 per cent.; β-naphthol, 5 per cent.; ichthyol, 5 per cent.; and iodoform, 2 per cent. They can be recommended to members of the profession who are desirous of using any of these drugs and combinations in the form of a soap.

